# Vaginal cuff dehiscence in laparoscopic hysterectomy: influence of various suturing methods of the vaginal vault

**DOI:** 10.1007/s10397-012-0745-5

**Published:** 2012-05-03

**Authors:** M. D. Blikkendaal, A. R. H. Twijnstra, S. C. L. Pacquee, J. P. T. Rhemrev, M. J. G. H. Smeets, C. D. de Kroon, F. W. Jansen

**Affiliations:** 1 Department of Gynaecology, Leiden University Medical Centre, PO Box 9600, 2300 RC Leiden, the Netherlands; 2 Department of Gynaecology, Bronovo Hospital, PO Box 96900, 2509 JH The Hague, the Netherlands

**Keywords:** Vaginal cuff dehiscence, Laparoscopic hysterectomy, Barbed suture, Laparoscopic suturing

## Abstract

Vaginal cuff dehiscence (VCD) is a severe adverse event and occurs more frequently after total laparoscopic hysterectomy (TLH) compared with abdominal and vaginal hysterectomy. The aim of this study is to compare the incidence of VCD after various suturing methods to close the vaginal vault. We conducted a retrospective cohort study. Patients who underwent TLH between January 2004 and May 2011 were enrolled. We compared the incidence of VCD after closure with transvaginal interrupted sutures versus laparoscopic interrupted sutures versus a laparoscopic single-layer running suture. The latter was either bidirectional barbed or a running vicryl suture with clips placed at each end commonly used in transanal endoscopic microsurgery. Three hundred thirty-one TLHs were included. In 75 (22.7 %), the vaginal vault was closed by transvaginal approach; in 90 (27.2 %), by laparoscopic interrupted sutures; and in 166 (50.2 %), by a laparoscopic running suture. Eight VCDs occurred: one (1.3 %) after transvaginal interrupted closure, three (3.3 %) after laparoscopic interrupted suturing and four (2.4 %) after a laparoscopic running suture was used (*p* = .707). With regard to the incidence of VCD, based on our data, neither a superiority of single-layer laparoscopic closure of the vaginal cuff with an unknotted running suture nor of the transvaginal and the laparoscopic interrupted suturing techniques could be demonstrated. We hypothesise that besides the suturing technique, other causes, such as the type and amount of coagulation used for colpotomy, may play a role in the increased risk of VCD after TLH.

## Background

Vaginal cuff dehiscence (VCD) after hysterectomy is an adverse event with potential severe morbidity. The incidence of VCD after total laparoscopic hysterectomy (TLH) varies between 0.3 and 3.1 % [1–7]. This is higher compared with the abdominal (AH) and vaginal (VH) approach [1, 8]. Since the continuous increment in the number of hysterectomies performed laparoscopically, the aetiology of VCD and explanations for its association with TLH have been subjected to research. Patient characteristics, such as smoking, diabetes, advanced age, radiation therapy and chronic steroid administration, next to precipitating factors such as sexual intercourse, postoperative cuff infection and/or hematoma and increased abdominal pressure (e.g. coughing, vomiting and straining at toilet) have been addressed with regard to their association with VCD [1, 9, 10]. Nevertheless, none of these factors are unique for TLH. Therefore, an explanation could very well be found in some specific procedural steps used to achieve a hysterectomy by laparoscopic approach. Some authors state that electrosurgical colpotomy, often used in TLH, is responsible for suboptimal vaginal cuff healing, due to tissue necrosis and prolonged devascularisation [11]. Recently, several studies compared the influence of various vaginal vault closure techniques on the incidence of VCD after TLH. Jeung et al. conducted the only prospective study on this topic and found no difference between laparoscopically sutured interrupted figures-of-eight versus knotted double-layer running sutures (1.6 and 0.8 %, respectively) [5]. On the other hand, Uccella et al. reported a threefold increased incidence associated with laparoscopic single-layer interrupted suturing compared with transvaginal closure with interrupted sutures (0.18 and 0.64 %, respectively) [7]. However, Siedhoff et al. compared a barbed running suture with other laparoscopic suturing techniques and found no VCDs in the barbed suture group versus a VCD rate of 3.1 % for other methods of closure [6]. Similarly, Einarsson et al. described a non-comparative cohort in which the vaginal cuff was closed with a barbed suture. An incidence of 0.6 % of the patients requiring vaginal cuff re-suturing was found [3].

Internationally, the aetiology of VCD is still a matter of concern. Either in its technique (TLH) as in the used technology (electrosurgical colpotomy and/or suturing method), an explanation could be found for the higher incidence of VCD. In our quest to further improve vaginal vault closure, we have been using various suturing methods. At first, we switched from transvaginal closure of the vaginal vault to laparoscopic closure with interrupted sutures. Thereafter, we started using running sutures: both barbed suturing and an unknotted running suturing technique with clips. To compare these methods, a power analysis indicated that we would have needed 1,349 cases in each arm to detect a desired reduction of 50 % in the VCD rate of 3.4 % [11] (80 % power, type I error 0.05). Since we regarded an adequately powered prospective study to be impossible to perform and given the need for more information, we conducted a retrospective cohort study based on prospectively collected data on this subject. This study aims to compare the incidence of vaginal cuff dehiscence with transvaginal closure of the vaginal vault versus laparoscopic closure with knotted interrupted sutures versus laparoscopic closure with two different unknotted single-layer running suturing methods.

## Methods

A university hospital (Leiden University Medical Centre, Leiden) and an affiliated teaching hospital (Bronovo Hospital, The Hague) participated in this study. All patients who underwent a TLH for benign and (pre)malignant indications between January 2004 and May 2011 were enrolled. Three gynaecologists (JPTR, MJGHS and FWJ) performed all procedures and used similar techniques and instruments over time. According to the surgeon's preference and availability, the procedures were performed by one or two surgeons. At the start of the study, all surgeons were already experienced in advanced laparoscopic surgery.

TLH was carried out similar to a recently described technique [12]. Briefly, all classic surgical steps are carried out laparoscopically, using bipolar energy for dissection of the ligaments and coagulation of the vascular pedicles. The bladder peritoneum is dissected with ultrasonic energy and the cervico-vaginal fascia is identified anteriorly. Hereafter, the sacro-uterine ligament is dissected posteriorly and the vaginal fornix is opened circularly using ultrasonic energy, while cranial traction with the uterine manipulator is provided. To the surgeon's preference, during this step (additional), bipolar energy is used as well. The vaginal cuff is sutured transvaginally (interrupted sutures with Vicryl no. 0, Ethicon, Johnson & Johnson Medical GmbH, Norderstedt, Germany) or laparoscopically (interrupted sutures or a running suture, both single-layer). In every stitch, a full thickness bite of approximately 1 cm is obtained, containing recto-vaginal fascia and vaginal mucosa posteriorly and vaginal mucosa and pubo-cervical fascia anteriorly. In laparoscopic closure of the vaginal vault, Vicryl no. 0 is used for the interrupted sutures, which are secured with intracorporeal tied knots. In case of a running suture, two different suturing methods are used according to the surgeon's preference. In one method, a double-armed barbed suture (Quill^TM^ Self-Retaining System; Angiotech Pharmaceuticals Inc., Vancouver, British Columbia, Canada) is used, in which the barbs change direction at mid-point. This suture is bidirectionally sutured from the midline to both lateral angles of the vaginal cuff [13]. In the other, we adopted (off label) a suturing technique commonly used in transanal endoscopic microsurgery (TEM). In this technique a regular Vicryl no. 0 with a suture staple placed at the distal end of the wire is sutured from the right to the left angle of the vaginal cuff, after which another suture staple is placed at the proximal end to secure the suture (suture clip forceps for TEM, Richard Wolf GmbH, Knittlingen, Germany). In all suturing methods, both utero-sacral ligaments are incorporated in the repair and the peritoneum is unclosed.

Patients were evaluated by anamnesis and physical examination 6 weeks postoperatively. Sexually active patients were instructed not to restart sexual intercourse until after this evaluation. All data were derived from a database supplemented by a chart review. For all patients, the type of suture (transvaginal interrupted, laparoscopic interrupted or laparoscopic running) was registered. Furthermore, patient characteristics (age, body mass index (BMI, in kilograms per square metre) and ASA classification) and procedure characteristics (operating time (in minutes, skin-to-skin), blood loss (in milliltre), uterus weight (in grams) and adverse outcomes) were obtained. Adverse events were registered for type of complication, severity (i.e. requiring re-intervention or not) and moment of onset, up to 6 weeks after discharge (i.e. marking the legitimate adverse event reporting period), according to the definitions and regulations as determined by the Guideline Adverse Events of the Dutch Society of Obstetricians and Gynaecologists [14].

The primary outcome was the incidence of VCD by type of suture (transvaginal interrupted (group 1) versus laparoscopic interrupted (group 2) versus laparoscopic running (group 3)). According to literature, we defined VCD as a partial or complete separation of the vaginal cuff that required surgical intervention, regardless of the presence of an open peritoneum and/or evisceration [1]. As a secondary assessment, we collected additional data of all these patients to identify possible characteristics associated with this complication. This included the trigger event to onset of dehiscence, presenting symptoms at the time of dehiscence, presence of an open peritoneum, presence of evisceration, type of repair, the interval time (in days) between TLH and dehiscence, relevant comorbidities (i.e. smoking, diabetes, use of immune suppressing drugs and radiotherapy), relevant accompanying complications (i.e. vaginal cuff cellulitis, infection or hematoma), indication for surgery, menopausal status, type of energy used for colpotomy (bipolar, ultrasonic or a combination) and use of prophylactic antibiotics at the time of hysterectomy. All procedures in which the vaginal cuff was sutured by conventional open approach (i.e. after conversion to laparotomy or after a minilaparotomy for specimen retrieval) were excluded.

To calculate differences between the groups, SPSS 17.0 statistical software (Chicago, IL, USA) was used. A Pearson chi-square test was used to compare proportions, and a one-way analysis of variance (ANOVA) was used for continuous variables. Pairwise *t* tests with Bonferroni's correction were used for post hoc multiple comparison. If the condition of a normal distribution (kurtosis between −1 and +2) was not met, additionally a Kruskal–Wallis test was performed to confirm the *p* value calculated by the ANOVA. *p* values <0.05 were considered statistically significant.

## Findings

During the study period, a total of 333 TLHs were performed. Of these, two procedures were converted to laparotomy. These two procedures were excluded from further analysis (no VCD reported). Finally, 331 TLHs were included in the analysis. In 75 patients (22.7 %), the vaginal vault was closed by transvaginal approach. Laparoscopic interrupted sutures were used for closure in 90 procedures (27.2 %), and a laparoscopic running suture was used in 166 procedures (50.2 %, 81 barbed sutures and 85 TEM sutures). The baseline characteristics of these three groups are detailed in Table 1. Compared with group 2, patients in group 1 had a lower ASA classification (*p* = .014), while blood loss was higher (*p* = .003). Compared with group 3, patients in group 1 had a lower BMI (*p* = .014), while blood loss was higher (*p* ≤ .001). This difference in blood loss is partly caused by two procedures in group 1 with an estimated blood loss of 2,300 and 950 mL, respectively (uterus weight 880 and 650 g, respectively; length of surgery 335 and 160 min, respectively). Nevertheless, after exclusion of these two statistical outliers, the differences in blood loss remained significant (mean blood loss in group 1, 188 mL; SD ±178 mL; *p* = .028 compared with group 2 and *p* = .002 compared with group 3). All other baseline characteristics were comparable between each group.

**Table 1:** Baseline characteristics of all procedures by suture method of the vaginal vault (transvaginal interrupted versus laparoscopic interrupted versus laparoscopic running) (*n* = 331)

	Group 1, transvaginal interrupted sutures (*n* = 75)		Group 2, laparoscopic interrupted sutures (*n* = 90)		Group 3, laparoscopic running sutures (*n* = 166)		ANOVA: Overall	Bonferroni: group 1 versus 2	Bonferroni: group 1 versus 3	Bonferroni: group 2 versus 3
	Mean ± SD	Range	Mean ± SD	Range	Mean ± SD	Range	*p* value	*p* value	*p* value	*p* value
Age (years)	47.2 ± 7.3	(32.5–66.1)	47.5 ± 8.5	(32.8–79.3)	49.0 ± 9.1	(29.0–78.3)	.230	–	–	–
BMI (kg/m^2^)	25.3 ± 3.6	(18.1–35.0)	26.3 ± 5.2	(16.2–44.1)	27.5 ± 6.1	(17.5–48.0)	.013	NS	.014	NS
ASA classification^a^	1^b^ ± 0.4	(1–2)	1^b^ ± 0.5	(1–3)	1^b^ ± 0.6	(1–3)	.018	.014	NS	NS
Length of surgery (min)^a^	141 ± 49	(60–335)	128 ± 32	(70–240)	129 ± 40.0	(50–260)	.082	–	–	–
Blood loss (mL)^a^	226 ± 312	(25–2300)	129 ± 148	(25–1,000)	120 ± 122	(25–800)	<.001	.003	<.001	NS
Uterus weight (g)	283 ± 181	(35–822)	228 ± 163	(35–700)	249 ± 197	(31–950)	.202	–	–	–

*SD* standard deviation, *NS* not significant

^a^ *p* value was confirmed by Kruskal–Wallis test because of a non-normal distribution

^b^Median

Overall, eight vaginal cuff dehiscences occurred: one (1.3 %) after transvaginal interrupted closure, three (3.3 %) after interrupted laparoscopic suturing and four (2.4 %) after a laparoscopic running suture was used (Table 2). There was no statistical difference with regard to VCD between these three groups (*p* = .707). In addition, we plotted all procedures in a consecutive order—separately for each surgeon—and marked the cases complicated by a VCD. These graphs showed that the VCDs did not tend to occur more frequently within the beginning period of each suturing method (not shown). Furthermore, the overall complication rate (regarding all severities) (20.0 versus 17.8 versus 13.3 %, *p* = .373) and the rate of complications requiring re-intervention (2.7 versus 3.3 versus 3.0 %, *p* = .773) were similar between the groups as well. In all but three patient records (99.1 %), both anamnesis and physical examination during the postoperative clinical evaluation after 6 weeks were clearly registered. Table 3 represents the characteristics of all patients that presented with a vaginal cuff dehiscence. Within the patient and procedure characteristics, no obvious predisposing factors could be identified. All patients received prophylactic antibiotics at time of hysterectomy. During all the procedures, ultrasonic energy and bipolar coagulation were alternately used for colpotomy and haemostasis. All eight patients presented with (heavy) vaginal blood loss. Two cases were (most likely) accompanied by another complication. In the first, an old vaginal vault haematoma appeared to be present during exploration in the operating room. In the last case, based on anamnesis and physical examination, sexual intercourse most likely caused an abscess to ‘spontaneously’ drain. In at least half of the cases, the patient had marked intercourse as the trigger event for the complaint; all presented with abdominal pain. In two cases a small dehiscence of the peritoneum was present. However, no evisceration occurred. In three patients, a vaginal cuff dehiscence occurred after the 6 weeks follow-up examination, on the 57th, 71st and 75th day, respectively, all after sexual intercourse. Except for one of these patients in which some granulation tissue was treated with silver nitrate, anamnesis and physical examination during the regular follow-up examination did not reveal other abnormalities in the postoperative course. One case was complicated by a fallopian tube prolapse. In this case, both the prolapse and the vaginal cuff dehiscence could be managed laparoscopically. In all other cases, vaginal (re)suturing of the dehiscence was sufficient. After repair, further recovery was uneventful in all eight patients.

**Table 2:** Incidence of vaginal cuff dehiscence and other complications by type of suture (*n* = 331)

	Group 1, transvaginal interrupted sutures (*n* = 75)	Group 2, laparoscopic interrupted sutures (*n* = 90)	Group 3, laparoscopic running sutures (*n* = 166)	*p* value
Vaginal cuff dehiscence (%)	1 (1.3)	3 (3.3)	4 (2.4)	.707
Overall complications (%)	15 (20.0)	16 (17.8)	22 (13.3)	.373
Requiring (re)intervention (%)	2 (2.7)	3 (3.3)	5 (3.0)	.773

**Table 3:** Characteristics of all patients with a vaginal cuff dehiscence

Case	Age (years)	BMI (kg/m^2^)	ASA	Length of surgery (min)	Blood loss (mL)	Uterus weight (g)	Indication for hysterectomy	Postmenopausal	Prophylactic antibiotics at hysterectomy	Suture type	Energy used for colpotomy	Trigger event	Presenting symptoms	Time after hysterectomy (days)	Peritoneum open	Evisceration	Type of repair	Relevant comorbidities	Relevant accompanying complications
1	55	35	2	135	100	Unknown	EC	Yes	Yes	Transvaginal interrupted	Bipolar and ultrasonic	Spontaneous	VBL	13	No	No	Transvaginal resuturing	None	Vaginal vault haematoma
2	41	30	1	115	150	Unknown	DUB and UM	No	Yes	Laparoscopic interrupted	Bipolar and ultrasonic	Spontaneous	VBL	15	No	No	Transvaginal suturing	Smoking	None
3	49	25	2	120	155	Unknown	DUB	No	Yes	Laparoscopic interrupted	Bipolar and ultrasonic	Spontaneous	VBL	20	No	No	Transvaginal suturing	None	None
4	56	24	1	105	25	100	EC	Yes	Yes	Laparoscopic interrupted	Bipolar and ultrasonic	Spontaneous	VBL	28	No	No	Transvaginal suturing	None	Granulation
5	46	23	1	110	50	315	UM	Yes	Yes	Laparoscopic running (Quill^TM^)	Bipolar and ultrasonic	Intercourse	VBL and pain	75	No	No	Transvaginal suturing	None	Granulation
6	40	26	1	125	25	360	UM	No	Yes	Laparoscopic running (Quill^TM^)	Bipolar and ultrasonic	Intercourse	VBL and pain	71	Yes	No	Laparoscopic resuturing	None	Fallopian tube prolapse
7	50	25	1	105	200	150	UM	No	Yes	Laparoscopic running (TEM)	Bipolar and ultrasonic	Intercourse	VBL and pain	57	Yes	No	Transvaginal suturing	None	None
8	34	22	1	95	75	140	UM	No	Yes	Laparoscopic running (TEM)	Bipolar and ultrasonic	Intercourse	VBL and pain	41	No	No	Transvaginal suturing	None	Abscess (most likely^a^)

*EC* endometrial cancer, *DUB* dysfunctional uterine bleeding, *UM* uterine myomas, *TEM* suture method adopted from transanal endoscopic microsurgery (see ‘Methods’ section), *VBL* vaginal blood loss

^a^Based on anamnesis and physical examination, this VCD most likely occurred after drainage of an abscess during sexual intercourse

## Discussion

VCD is a potentially severe adverse event. Internationally, the reason for the increased incidence of VCD after TLH is still a matter of concern. The used suturing method of the vaginal vault is mentioned as an aetiological factor. In our comparison of laparoscopic suturing of the vaginal cuff with a single-layer unknotted running suture and both laparoscopic and transvaginal closure with knotted interrupted sutures, we found the lowest incidence of VCD after transvaginal suturing (1.3 %). This was followed by both the barbed suture and the running vicryl suture with TEM clips (2.4 %), which proved to be an easy to adopt alternative. However, based on our data, no statistical superiority of either of these suturing methods could be proven. Regardless of these suturing techniques, the incidence of VCD after TLH remains high compared with abdominal and vaginal hysterectomy. Therefore, other steps of the procedure unique to TLH, such as the amount and type of coagulation used for colpotomy, should be assessed in future research as possible determinants for the onset of VCD.

To our knowledge, the present study is the first to compare single-layer running suturing techniques with interrupted sutures for closure of the vaginal cuff. Additionally, cuff closure using a running vicryl suture with TEM clips is a newly introduced alternative to other suturing techniques currently in use. The safety and effectiveness of barded sutures already has been demonstrated in two other studies [3, 6]. However, one was non-comparative and in the other a more time-consuming double-layer suturing method was used. Furthermore, the barbed suture proved to be relatively easy to learn [6]. In our experience as well, both the single-layer barbed suture and the single-layer running vicryl suture with TEM clips proved to be easy to adopt and as safe—regarding incidence of VCD—as transvaginal and laparoscopic closure of the vaginal cuff with interrupted sutures.

Both techniques allow laparoscopic closure of the vaginal vault to be less time-consuming, due to their unknotted fashion. However, some concern is expressed regarding adhesion formation of the intestine to the tail of the barbed suture, which in turn potentially could cause bowel obstruction [15–17].

As shown in Table 1, due to the retrospective design of our study, some differences in the baseline characteristics occurred. Especially with regard to the aetiology of VCD, the observed differences in mean BMI and mean intraoperative blood loss are, however, not clinically relevant. Furthermore, the same counts for the difference in ASA classification between group 1 and group 2, since none of the patients presenting with a VCD suffered from a systemic disease which potentially could induce this complication (e.g. diabetes or chronic cough due to chronic obstructive pulmonary disease). Finally, given the relatively long study period (in which the same surgical techniques and instruments were used), we had to rule out a possible influence of surgical experience to explain these differences. However, near the end of the study period, VCD tended to occur as (in)frequent as at the beginning.

VCD is still a matter of concern to those who perform TLH. Although techniques for suturing of the vaginal cuff have changed rapidly over the past years, only one prospective study on this subject has been published [5]. It compared laparoscopic closure with interrupted and running sutures, however, with a double-layer suturing method and with an extracorporeal knotting technique. Recently, Uccella et al. advocated a superiority of transvaginal closure based on data of their own retrospective cohort and a review of literature in which they found a threefold increase in the incidence of VCD associated with laparoscopic closure [7]. Our study suggests a similar difference between transvaginal closure and laparoscopic closure with knotted interrupted sutures. However, they did not compare the use of laparoscopic running suturing methods. Given the fact that transvaginal closure cannot always be accomplished in all women, alternatives to this suturing method should be studied. Unfortunately, a prospective intention-to-treat study to test this superiority will be hard to perform. Based on a pooled incidence of 0.18 % [7] (transvaginal closure) versus 2.4 % (laparoscopic running unknotted suture, present study), we measured that at least 405 patients should be included in each arm to obtain adequate power (two-sided test for independent samples with 80 % power and 5 % type I error). To ensure that the same surgical technique is applied in all procedures, ideally, a single-centre study needs to be conducted. As a result, the conclusions drawn from the present study have to be strengthened by pooling of data with future publications on this topic.

Several explanations why hysterectomy by laparoscopic approach is prone to have a higher rate of VCD have been put forward. Firstly, regarding initial sexual intercourse as a precipitating event, it has been suggested that the rapid recovery after the laparoscopic approach, compared with the abdominal approach, facilitates swift return to everyday activities and early resumption of (sexual) activities, which could predispose rupture of the vaginal vault [10, 18]. On the other hand, this assertion does not seem to hold, whereas also in our study most VCDs related to intercourse occurred after the regular 6 weeks postoperative follow-up examination, which is considered to be sufficient time for primary wound healing [9–11, 18–20].

Secondly, several studies suggested that the amount and type of energy used for colpotomy could be predisposing for VCD [5, 18, 21, 22]. Gruber et al. performed a histopathologic assessment to compare the thermal damage after the use of ultrasonic, monopolar and bipolar energy for colpotomy in swine. They concluded that ultrasonic energy causes the least and bipolar energy the greatest tissue damage [21]. In all our procedures, including those complicated by a VCD, ultrasonic energy was used for colpotomy and additional bipolar energy was used for haemostasis (Table 3). The amount of coagulation used in the cases in which a VCD occurred compared with the procedures after which no VCD occurred is, however, unclear. Nevertheless, in order to maintain sufficient vascularisation, minimising the use of bipolar energy for haemostasis seems advisable. Preferably, only arterial bleeders should be coagulated and one should rely on the sutures to control venous oozing. This recommendation is supported by the lower reported incidence of VCD after conventional abdominal approach to hysterectomy, in which the vaginal vault is clamped and sutured and no coagulation is used on a regular basis [23].

Furthermore, several studies did address the type and class of suture material as a possible cause for vaginal cuff dehiscence [11, 19, 22]. However, review of the literature yields neither evidence nor consensus on the preferred suture material, concerning monofilament versus multifilament and delayed absorbability of the thread.

Finally, surgical characteristics such as the technical difficulty of laparoscopic surgery, the high complexity of laparoscopic knot tying and insufficient amounts of tissue incorporated in the suture have been suggested as reasons for the increased incidence of VCD in LH [5–7, 13]. The placement of sutures in ‘big bites’ of viable tissue seems justified [5, 18].

It is more likely that a VCD occurs secondary to an underlying factor such as a haematoma or a primary healing defect as a result of excessive coagulation. Hypothetically, in these cases, the vaginal wall epithelium remains approximated only by the suture. Therefore, as soon as the suture loses most of its tensile strength, a (partial) separation of the vaginal cuff occurs. This hypothesis is supported by the difference in days between surgery and VCD, which we found in the present study (Table 3). With regard to the barbed suture (*n* = 2), the mean time to VCD was 73 days. For the other suturing methods (*n* = 6), in which regular Vicryl no. 0 was used, the mean time to VCD was 29 days. This difference can be explained by the fact that the tensile strength of Vicryl is 25 % after 4 weeks (http://www.ecatalog.ethicon.com/sutures-absorbable), whereas the tensile strength of the barbed suture is still 80 % [6]. Sexual intercourse might only trigger breakdown of a partially dissolved suture, which in case of such a primary healing defect, causes a (partial) separation of the vaginal wall epithelium that would have occurred sooner or later anyway. In our opinion, the advice to refrain from intercourse up to 3 months after TLH, as suggested by others, is neither based on the pathophysiological process of VCD nor based on evidence [2, 24]. Similarly, given the ambiguous relationship of intercourse and VCD, we thus tend to emphasise to our patients that from a clinical point of view they themselves are not to blame for this embarrassing event.

The VCD rate of 3.3 % that we found for laparoscopic interrupted sutures was relatively high but was similar to the rate published by others before they started to use the barbed suture [6]. However, more importantly, in these cases the peritoneum remained closed and in none (of all our cases) an evisceration occurred. Especially the latter is important, since immediate reoperation is needed and its association with bowel perforation and/or necrosis, peritonitis and general sepsis [7, 9, 25].

## Conclusion

In conclusion, based on our data, no superiority of one of the suturing methods over the other was found and the exact aetiology of VCD still remains unclear. Regardless of the suturing method, we hypothesise that the surgical approach towards the colpotomy in TLH in comparison to the abdominal approach, with additional (extensive) application of coagulation, has inherent its specific side effects. To enable future scientific analysis of pooled data, we would like to challenge others to publish their data and opinion on this important subject.
